# Differential effects of yeast derivatives on pepper productivity, stress resilience, and soil microbial communities

**DOI:** 10.7717/peerj.21604

**Published:** 2026-10-02

**Authors:** Siwei Liang, Guogeng Jia, Hui Chen, Can Zhou, Wenjie Peng, Shuxing Liu, Chao Ai, Yan Zhang, Guangda Ding

**Affiliations:** 1 National Key Laboratory of Agricultural Microbiology, Hubei Hongshan Laboratory, Huazhong Agricultural University, Wuhan, China; 2 Angel Yeast Co., Ltd, Yichang, China; 3 The Institute of Agricultural Resources and Regional Planning, Chinese Academy of Agricultural Sciences, Beijing, China; 4 College of Resources and Environment, Huazhong Agricultural University, Wuhan, China

**Keywords:** Yeast derivatives, *Capsicum annuum L.*, Nutrient use efficiency, Low-temperature stress tolerance, Rhizosphere microbial communities

## Abstract

The excessive application of chemical fertilizers is a prevalent issue in modern agriculture, as it increases production costs, undermines economic returns, and degrades soil health. In this study, the effects of two compositionally distinct yeast derivatives, namely Yeast Glycoside Type I (YD1) and Water-Soluble Yeast Glycoside (YD2), on the growth, nutrient use efficiency, low-temperature (LT) stress tolerance, and rhizosphere microbial communities of pepper (*Capsicum annuum* L.) were investigated. The yeast derivatives (YD) were characterized using scanning electron microscopy (SEM) and biochemical analysis, which revealed distinct profiles: YD2 contained higher nitrogen (11.89%), isindole-3-acetic acid (IAA), and 1-Aminocyclopropane-1-carboxylic acid (ACC), whereas YD1 was enriched in cytokinins (isopentenyl adenine, iP; trans-zeatin, tZ). Under normal temperature (NT) conditions, application of YD2 at concentrations of 1% significantly enhanced pepper yield, shoot biomass, and nitrogen/potassium use efficiency, whereas YD1 exerted minimal effects. However, under low-temperature (LT) stress, applications of YD1 and YD2 did not significantly increase yield. Despite this, YD2 maintained root activity, mitigated chlorophyll degradation, and increased the activities of antioxidant enzymes under LT stress. Both YD altered rhizosphere bacterial community structure under NT conditions (PC1: 42.09%, *p* = 0.007); however, this effect was attenuated under LT stress (PC1: 24.85%, *p* = 0.003). Specifically, YD1 was associated with an increased relative abundance of * Microscillaceae* (NT) and * Thiobacillus* (LT), while YD2 was linked to an increase in * Kaiserbacteria* (NT) and * Armatimonadales* (LT). These findings suggest that the two YDs, particularly YD2, can influence pepper productivity and stress resilience through associations with improved nutrient uptake, physiological adjustments, and shifts in the rhizosphere microbiome. This pot-based study provides insights into the potential of tailored yeast derivatives as biostimulants, offering a basis for developing more sustainable agricultural practices.

## Introduction

By 2050, the global population is projected to reach 9.7 billion, with a further increase to 10.9 billion by 2100 (Gu, Andreev & Dupre, 2021). To meet escalating demand for food, modern agricultural systems have become increasingly dependent on chemical fertilizers. However, these systems face substantial challenges due to the overuse of chemical fertilizers—a practice that has become prevalent in efforts to maximize crop yields. China, which supports 20% of the world’s population with only 7% of the global arable land, consumes more chemical fertilizers than any other country globally (Smith & Siciliano, 2015; Sun et al., 2012). Notably, the average nutrient use efficiency of crops in China remains low: merely 33% for nitrogen (N), 24% for phosphorus (P), and 42% for potassium (K) (Tang et al., 2025). To address these pressing issues, there is an urgent need to develop and adopt sustainable agricultural practices that enhance fertilizer use efficiency and improve soil health. One promising alternative is the application of fertilizer synergists, which we define here as amendments that, when combined with conventional fertilizers, enhance nutrient use efficiency and/or plant stress tolerance beyond the additive effects of the individual components (Wang et al., 2023; Li et al., 2023).

Since its first isolation and identification in 1680, yeast has been widely used in various industries (Mukherjee et al., 2020). In agriculture, yeast-based products, including live yeast cells, yeast extracts, hydrolysates, and purified metabolites, can exhibit plant growth-promoting properties. It is important to distinguish among these forms, as their modes of action may differ. This study focuses on enzymatically hydrolyzed yeast derivatives, which contain a complex mixture of nutrients, cell wall components, and phytohormones. The direct mechanisms involve the provision or stimulation of phytohormones like indole-3-acetic acid (IAA) and cytokinins, and the enhancement of nutrient availability (Hashem, El-Homosy & El-Homosy, 2017; Nassar, El-Tarabily & Sivasithamparam, 2005). Indirectly, they may enhance plant resistance to stresses (Fu et al., 2016; Nassar, El-Tarabily & Sivasithamparam, 2005).

While the general benefits of yeast-based products are recognized, critical knowledge gaps persist. First, their efficacy as fertilizer synergists specifically for chili pepper (*Capsicum annuum* L.) cultivation is not well quantified. Second, their potential to mitigate low-temperature stress—a major constraint for pepper production—remains largely unexplored. Third, and crucially, there is a lack of understanding of how yeast derivatives with distinct biochemical compositions differentially influence plant performance. To address these gaps, we selected two commercially available (YD) (YD1 and YD2) for comparison. Preliminary analysis revealed that YD2 and YD1 possess divergent nutrient and phytohormone profiles (*e.g.*, YD2 is richer in nitrogen, IAA, and ACC; YD1 is richer in cytokinins like iP and tZ), providing a basis to test whether compositional differences lead to distinct agronomic and physiological outcomes.

Low-temperature stress was selected as the abiotic stress treatment for this study for several reasons. First, chili pepper (*Capsicum annuum* L.) is a warm-season crop highly sensitive to chilling temperatures, and low-temperature stress is a major constraint limiting pepper production in temperate and subtropical regions, particularly during early spring and autumn cultivation. Second, compared to other abiotic stresses such as drought or high temperature, low-temperature stress during the seedling and flowering stages is a particularly common and economically damaging challenge for pepper growers in many production areas (*e.g.*, China’s main pepper-producing regions). Third, the physiological mechanisms underlying low-temperature stress responses—including membrane damage, oxidative stress, and inhibition of photosynthesis—are well-characterized in pepper, providing a robust framework for evaluating the protective effects of biostimulants.

Therefore, this study aimed to: (1) evaluate and compare the effects of YD1 and YD2 on pepper growth, yield, and nitrogen (N), phosphorus (P), and potassium (K) use efficiency under normal temperature conditions; (2) assess their capacity to alleviate low-temperature stress by examining key physiological and biochemical responses; and (3) characterize their influence on the structure of the rhizosphere microbial community under both temperature regimes. We hypothesized that YD2, due to its higher auxin and ACC content, would be more effective than YD1 in promoting nutrient uptake and yield under normal conditions and in sustaining physiological function under cold stress. We further hypothesized that both YDs would alter the rhizosphere microbiome, but that this effect would be modulated by temperature.

## Materials and Methods

### YD characterization analysis

Yeast Glycoside Type I (YD1) and Water-Soluble Yeast Glycoside (YD2) were provided by Angel Yeast Co., Ltd. Both products were prepared from the yeast cream of *Saccharomyces cerevisiae* through autolysis and enzymatic hydrolysis, followed by evaporation, concentration, and drying. Notably, YD2 underwent two rounds of enzymatic hydrolysis to enhance its water solubility and active component release. The surface micromorphology of two YDs was observed *via* SEM (SU8010, Hitachi). Dry powder samples were fixed on aluminum stubs with conductive carbon tape, then sputter-coated with a ∼10 nm gold-palladium layer for better conductivity. SEM imaging was conducted at 1.0 kV high vacuum. Total N, P and K contents were determined by the Kjeldahl digestion–distillation method (Quirino et al., 2023), molybdenum-blue colorimetry (Angelova et al., 2022) and flame photometry (Ullah et al., 2022), respectively. Endogenous phytohormones were quantified by ultra performance liquid chromatography-tandem mass spectrometry (UPLC-MS/MS) with reference to a modified published method. Briefly, freeze-dried YD powder was extracted with methanol/water/formic acid mixed solution containing internal standards. After centrifugation and filtration, the supernatant was analyzed. Chromatographic separation was carried out on a BEH C18 column *via* gradient elution, and mass spectrometry detection was performed in MRM mode. Target phytohormones were quantified using external calibration curves with internal standard correction (Balcke et al., 2012).

### Fertilizer efficiency and dose optimization analysis

A pot-culture experiment with pepper was carried out in 22 cm-inner-diameter × 27 cm-height pots filled with a substrate of peat: perlite: vermiculite (7: 2: 1, v/v). Seeds of pepper (*Capsicum annuum* L.) cultivar ‘Luosijiao’ (commonly known as ‘Screw Pepper’) were used. Prior to sowing, seeds were soaked in warm water (30 °C) for 2–4 h and then germinated on moist sterile gauze in a constant temperature incubator at 30 °C. The pot experiment was conducted in a climate-controlled greenhouse at the Beijing Academy of Agriculture and Forestry Sciences (BAAFS), Beijing, China. After transplanting at the four-leaf stage, pepper seedlings were cultivated under the following environmental conditions before temperature treatment initiation: a photoperiod of 14/10 h (light/dark), the photosynthetically active radiation (PAR) at the canopy level maintained at approximately 600 ± 50 µmol m^−2^ s^−1^ *via* natural sunlight combined with supplemental LED lighting, a day/night temperature regime of 28 °C /22 °C, and relative humidity ranging from 60% to 80%.

The baseline fertilization rate was 600 kg N ha^−1^, 300 kg P_2_O_5_ ha^−1^, and 840 kg K_2_O ha^−1^, giving a total NPK input of 1,740 kg ha^−1^. Two yeast derivatives (YD1 and YD2) were evaluated at five substitution rates: 0%, 0.5%, 1%, 2.5%, and 5% (w/w) of the total NPK weight. These treatments are designated YD-0, YD−0.5, YD-1, YD−2.5, and YD-5, respectively. The YD powders were thoroughly mixed with the corresponding chemical fertilizer at the specified rates (0%, 0.5%, 1%, 2.5%, or 5% of the total NPK weight). This YD-fertilizer mixture was then dissolved in an appropriate volume of water to form a liquid solution. The solution was applied to the potting substrate *via* root drenching (irrigation) at the designated growth stages. Three pots were repeated for each treatment.

Pot-level application rates were calculated based on a planting density of 45,000 plants ha^−1^. The amount of chemical fertilizer and YD applied per pot (containing one plant) for each treatment.

Nutrient use efficiency (NUE) was calculated accounting for the total nutrient input from both the substituted chemical fertilizer and the applied YD. Aboveground plant tissues were analyzed for N, P, and K concentration. Nutrient uptake (U, mg plant^−1^) was calculated as biomass multiplied by nutrient concentration.

On a per-plant basis, fertilizer rates were calculated according to the 45,000 plants ha^−1^ density; 40% of the nutrients were applied from pre-flowering to first flowering (20% incorporated into the substrate as basal dressing and 20% top-dressed in two equal splits), while the remaining 60% was supplied in four equal fertigation between full flowering and physiological maturity.

### Effects of YDs on physiological indicators and root hormones in pepper under low temperature stress

Pepper cultivation was performed as described above. After 45 d, temperature treatments consisted of normal temperature (NT: 28 °C/22 °C, day/night) and low temperature (LT: 16 °C/10 °C, day/night). Fertilization treatments included a basal fertilizer amended with either 1% YD1 or 1% YD2. All fertilizer treatments received the same total nutrient supply; corresponding unfertilized controls were included for comparison. Plants were sampled 15 days after treatment initiation. Root activity, reflecting dehydrogenase activity as an indicator of metabolic vigor, was measured using a modified triphenyltetrazolium chloride (TTC) reduction assay adapted from Clemensson-Lindell (1994); Chlorophyll was extracted and quantified following the method of Ngcobo et al. (2024). Fresh leaf tissue was ground in a mortar with 10 mL of an 80% (v/v) acetone-ethanol (1:1) mixture containing a small amount of calcium carbonate. The homogenate was filtered, and the residue was re-extracted until colorless. The combined filtrate was made up to a final volume of 25 mL with the extraction solvent. Absorbance of the extract was measured at 645 nm and 663 nm using a spectrophotometer. Chlorophyll a, chlorophyll b, and total chlorophyll concentrations (mg g^−1^ fresh weight) were calculated using the following equations:

Chlorophyll a (mg g^−1^ FW) = [(12.7 × A_6__6__3_)−(2.69 × A_6__4__5_)] × (V/(1,000 × W))

Chlorophyll b (mg g^−1^ FW) = [(22.9 × A_6__4__5_)−(4.68 × A_6__6__3_)] × (V/(1,000 × W))

Total chlorophyll = Chlorophyll a + Chlorophyll b

where A is absorbance, V is the final extract volume (mL), and W is the fresh weight of the sample (g).

Proline content was determined using the ninhydrin-based method described by Carillo & Gibon (2011). Fresh leaf tissue (0.5 g) was homogenized in five mL of 3% (w/v) sulfosalicylic acid. The homogenate was centrifuged at 10,000× g for 10 min. Two mL of the supernatant was reacted with two mL of acid ninhydrin reagent (1.25 g ninhydrin in 30 mL glacial acetic acid and 20 mL 6 M phosphoric acid) and 2 mL of glacial acetic acid in a boiling water bath for 1 h. The reaction was terminated in an ice bath. The chromophore was extracted with four mL of toluene, and its absorbance was read at 520 nm. Proline concentration was calculated using a standard curve of L-proline and expressed as micrograms per gram fresh weight (µg g^−1^ FW). Soluble sugars were quantified using the anthrone sulfuric acid method (Zhang, Zong & Huang, 2020). Fresh leaf tissue (0.2 g) was homogenized in five mL of distilled water and incubated in a boiling water bath for 30 min. After centrifugation at 5,000× g for 10 min, 0.5 mL of the supernatant was mixed with three mL of freshly prepared anthrone reagent (200 mg anthrone in 100 mL concentrated sulfuric acid). The mixture was heated in a boiling water bath for 10 min, cooled, and the absorbance measured at 620 nm. Glucose was used as a standard, and soluble sugar content was expressed as milligrams of glucose equivalents per gram fresh weight. Superoxide dismutase (SOD) activity: SOD activity was determined using the nitroblue tetrazolium (NBT) photoreduction method according to Alici & Arabaci (2016) with modifications. Fresh leaf tissue (0.5 g) was homogenized in five mL of ice-cold 50 mM phosphate buffer (pH 7.8) containing one mM ethylenediaminetetraacetic acid (EDTA) and 1% (w/v) polyvinylpyrrolidone (PVP). The homogenate was centrifuged at 12,000 × g for 15 min at 4 °C, and the supernatant was used as the crude enzyme extract. The reaction mixture contained 50 mM phosphate buffer (pH 7.8), 13 mM methionine, 75 µM NBT, 0.1 mM EDTA, 2 µM riboflavin, and 50 µL of enzyme extract. One set of tubes was illuminated under fluorescent light (4,000 lux) for 20 min, while another set was kept in the dark as a control. The absorbance of the illuminated samples was read at 560 nm against the dark control. One unit of SOD activity was defined as the amount of enzyme required to inhibit 50% of NBT photoreduction and was expressed as units per gram fresh weight (U g^−1^ FW). Peroxidase (POD) activity: POD activity was assayed using the guaiacol oxidation method (Alici & Arabaci, 2016). Fresh leaf tissue (0.5 g) was homogenized in five mL of 50 mM phosphate buffer (pH 6.5). The homogenate was centrifuged at 10,000 × g for 15 min at 4 °C. The reaction mixture (three mL) contained 50 mM phosphate buffer (pH 6.5), 20 mM guaiacol, 40 mM H_2_O_2_, and 50 µL of enzyme extract. The increase in absorbance due to the formation of tetraguaiacol was recorded at 470 nm for 2 min. Enzyme activity was calculated using the extinction coefficient of tetraguaiacol (26.6 mM^−1^ cm^−1^) and expressed as micromoles of tetraguaiacol formed per minute per gram fresh weight (µmol min^−1^ g^−1^ FW). For all physiological and biochemical analyses, destructive sampling was conducted 15 days after the initiation of temperature treatments. Each experimental replicate (*n* = 3) consisted of plant material pooled from three randomly selected pots within the same treatment.

### Effects of YDs on rhizosphere microbial community of pepper

The experimental design followed the same protocol as described in ‘Effects of YDs on physiological indicators and root hormones in pepper under low temperature stress’. Rhizosphere soil samples were collected at 15 days post-treatment under controlled conditions to ensure sample integrity. Immediately after collection, the samples were placed in sterile cryopreservation tubes, flash-frozen in liquid N to preserve microbial community structures and subsequently stored at −80 °C to prevent any biochemical alterations. Rhizosphere soil DNA was extracted, and the bacterial 16S rRNA gene amplicon sequencing was conducted by Majorbio Bio-Pharm Technology Co., Ltd. (Shanghai, China). Paired-end sequencing (2 × 250 bp) was performed on an Illumina NovaSeq 6000 platform. Raw data processing was performed on the Majorbio Cloud Platform. Briefly, reads were quality-filtered, merged, and chimera-checked. Amplicon Sequence Variants (ASVs) were generated using the divisive amplicon denoising algorithm 2 (DADA2) algorithm within the quantitative insights into microbial ecology 2 (QIME2) environment.

### Statistical analysis

All data were expressed as mean ± standard deviation (SD). Statistical analyses were performed using Origin Pro 2024. ANOVA analysis was applied. Tukey’s honest significant difference (HSD) test was adopted for *post-hoc* multiple comparisons. Significance was set at *p* < 0.05, and non-parametric tests were used for non-normal data.

## Results

### Characterization of two YDs

The results showed that YD1 contained 5.41% total N and 1.03% total P, whereas its K content was relatively low at 1.43%. In contrast, YD2 exhibited a much higher N content of 11.89%, with total P and K reaching 1.61% and 3.08%, respectively. Quantitative profiling of 13 phytohormones in the two YDs revealed distinct hormonal profiles. Overall, YD2 contained higher concentrations of indole-3-acetic acid (IAA), isopentenyladenosine (iPR) and 1-aminocyclopropane-1-carboxylic acid (ACC) than YD1; notably, the IAA concentration in YD2 was approximately ten times that in YD1. In contrast, YD1 showed greater abundance of the cytokinins isopentenyladenine (iP) and trans-zeatin (tZ). Jasmonic acid (JA) was detected exclusively in YD2, whereas it was below the limit of detection in YD1. Likewise, salicylic acid (SA) was more abundant in YD2. For the remaining phytohormones—including dihydrozeatin (DHZ), brassinolide (BL), castasterone (CS) and typhasterol (TY)—no marked differences were observed between the two YDs, and their absolute concentrations were relatively low (Table 1). In summary, YD2 is characterized as a derivative rich in auxins and ethylene precursors, whereas YD1 is predominantly enriched in cytokinins (iP and tZ).

**Table 1:** Phytohormone in YD.

**Types**	**YD1 (ng/g)**	**YD2 (ng/g)**
Jasmonic acid (JA)	–	32 ± 17.52
IAA	3,601.33 ± 339.02	36,664.33 ± 3376.2
SA	104.33 ± 33.38	344 ± 50.32
Dihydrozeatin (DHZ)	10 ± 1	11.67 ± 0.58
Isopentenyladenine (iP)	520.67 ± 61.4	122.67 ± 9.87
Isopentenyladenine riboside (iPR)	142.33 ± 4.51	2,266.67 ± 196.45
Trans-zeatin (tZ)	245.33 ± 13.32	31 ± 4
Cis-zeatin (cZ)	13 ± 1	3 ± 0
Trans-zeatin riboside (tzR)	0.67 ± 0.58	1.67 ± 0.58
Cis-zeatin riboside (czR)	–	2 ± 0
1-Aminocyclopropane-1-carboxylic acid (ACC)	135.33 ± 14.29	344 ± 17.35
Brassinolide (BL)	10.33 ± 3.21	6 ± 4.36
Castasterone (CS)	4.67 ± 0.58	2.67 ± 0.58
Typhasterol (TY)	12.33 ± 2.08	12.67 ± 1.53

SEM observation revealed distinct surface architectures between the two YDs. YD1 appeared as irregular, bulky fragments, whereas YD2 retained numerous intact or semiintact yeast cell residues (Fig. 1). While the YDs were dissolved in water prior to root drench application—and thus their solid-state morphology may not directly reflect their solution-state behavior—the SEM images were presented as a qualitative characterization of the raw material form of the two derivatives. They serve to visually document the physical differences resulting from distinct manufacturing processes, providing descriptive context for the compositional differences reported in Table 1. These images as descriptive observations rather than as mechanistic evidence.

**Figure 1:**
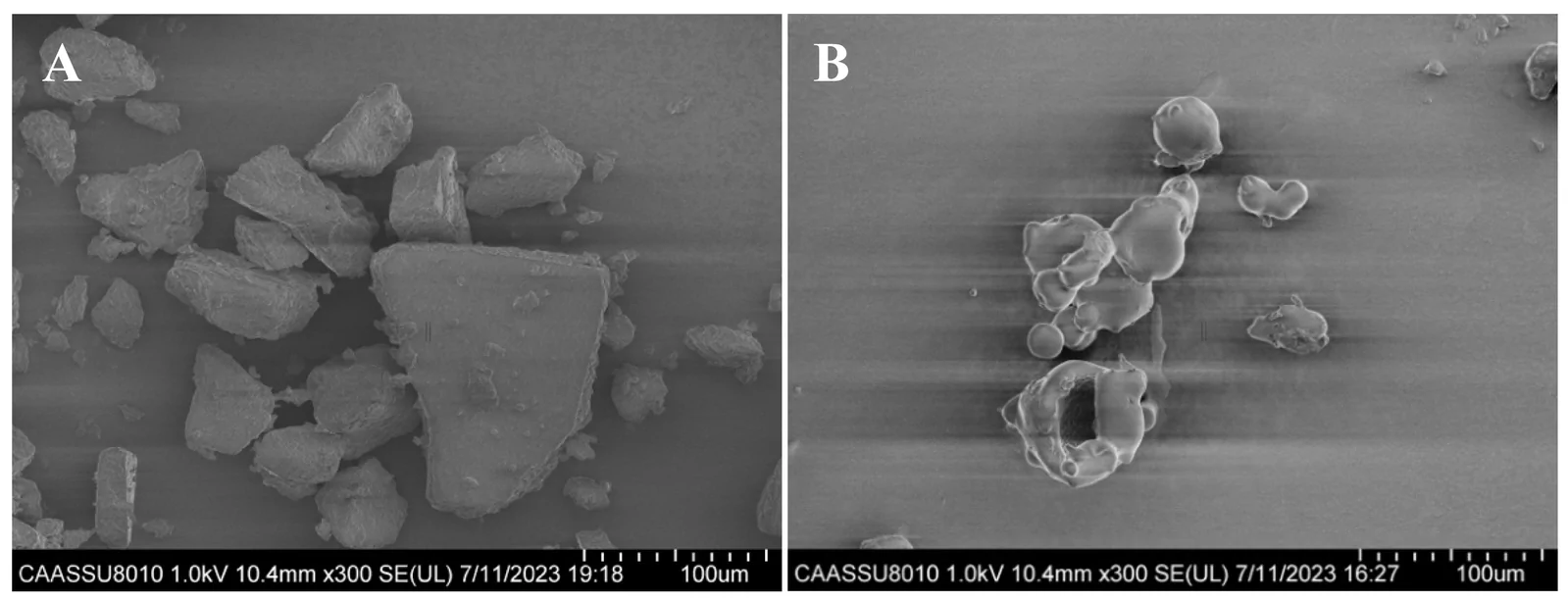
The SEM image of YDs. (A) Yeast derivatives 1, (B) Yeast derivatives 2.

### Effect of YD on pepper biomass and NPK utilization efficiency

Under normal temperature (NT) conditions, only application of YD at a 1% rate significantly increased pepper yield compared to the full-chemical-fertilizer control (YD-0). Moreover, there is no difference between these two derivatives. In contrast, under low-temperature (LT) stress, application of either YD1 or YD2 across a range of concentrations (0.5% to 5%) did not significantly increase pepper yield compared to the stressed control (Fig. 2). Furthermore, the application of YD1 did not significantly affect the weight of stem, leaves and roots compare with the YD-0 (Fig. 3). Conversely, high application rates of YD1, particularly 2.5%, showed a trend towards inhibiting root growth compared to the 1% application rate, with this inhibition being statistically significant (Fig. 3B). In contrast, the application of YD2 concentration of 1% significantly enhanced the yield of chili peppers. Notably, an application rate of 0.5% resulted in a significantly higher shoot biomass and root weight of chili peppers compared to the treatment with full chemical fertilizer and other application rates of YD2 (Fig. 3).

**Figure 2:**
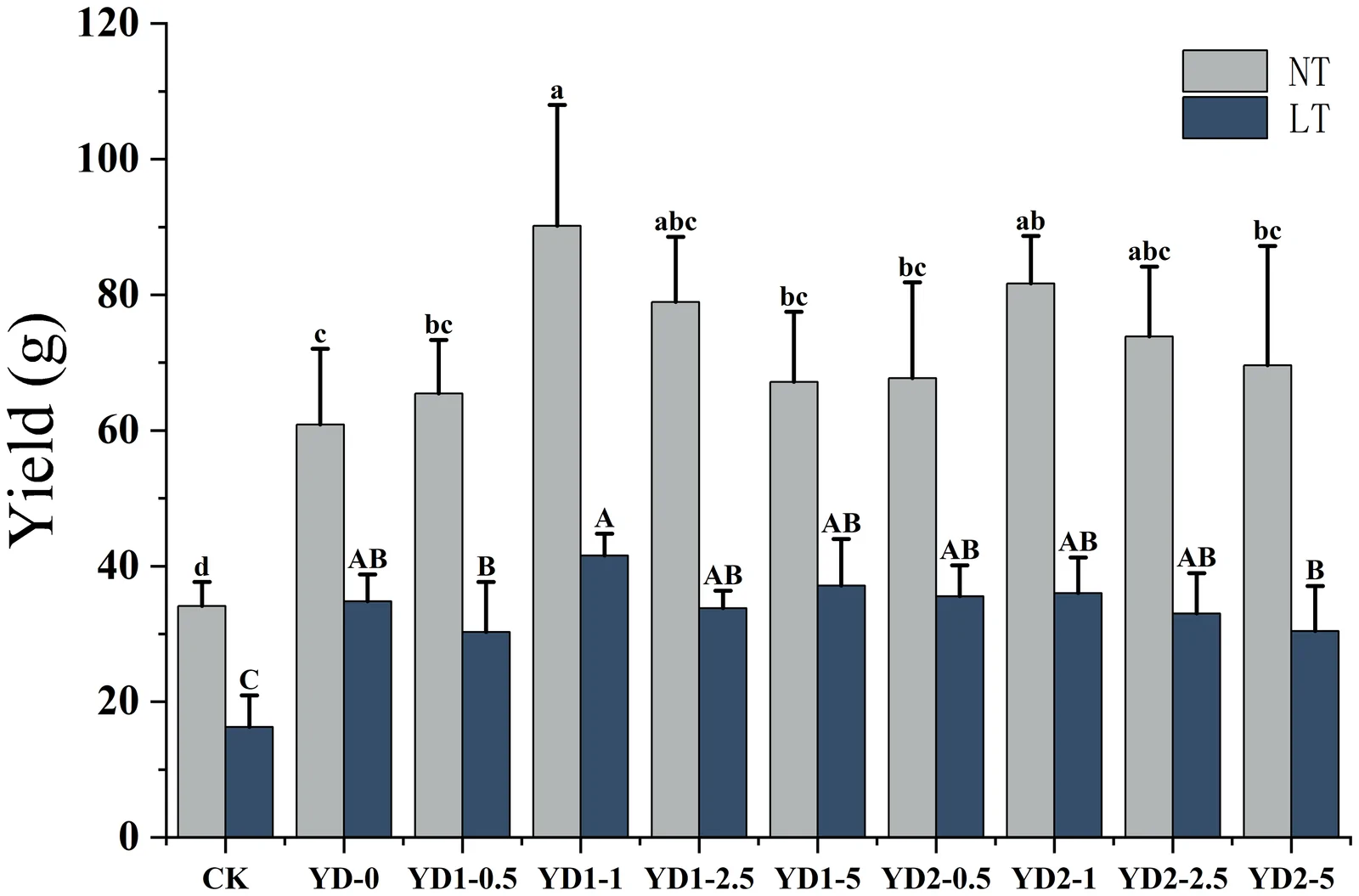
The yield of chili pepper under normal and low temperature conditions. NT, normal temperature; LT, low temperature.

**Figure 3:**
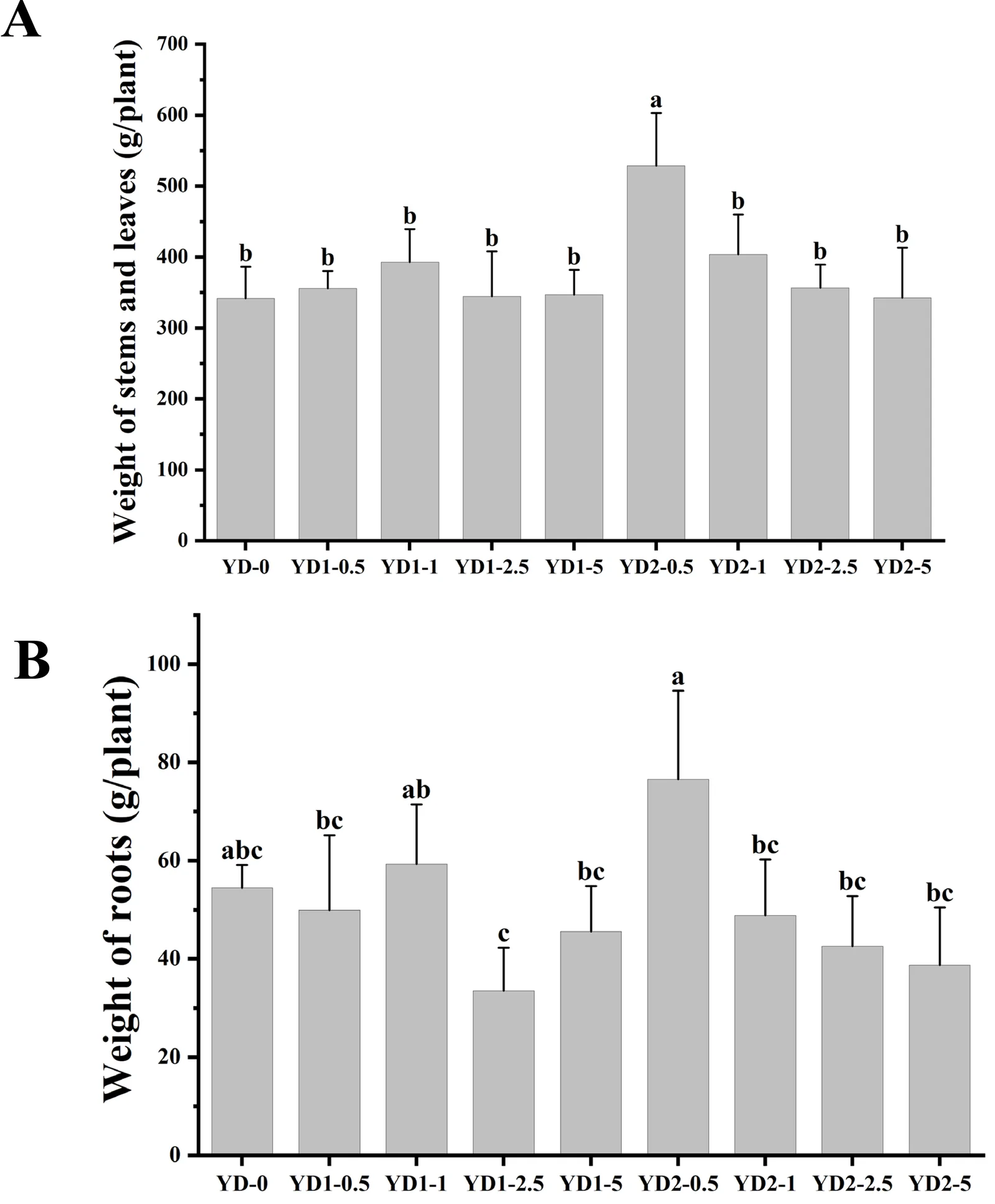
Effect of YDs on the biomass of pepper leaves, stems, and roots. (A) weight of stem and leaves per plant, (B) weight of roots per plant.

Under controlled pot conditions, the effects of two YDs on N, P and K use efficiencies of pepper were evaluated across five application rates (0.5%, 1%, 2.5% and 5%, v/v) compared to untreated control (YD-0). N use efficiency ranged from 36.98% to 65.34% (Fig. 4A), P use efficiency from 14.02% to 20.72% (Fig. 4B), and K use efficiency from 42.31% to 82.90% (Fig. 4C). YD1 application had no significant impact on pepper N, P, or K use efficiencies (Fig. 4). In contrast, YD2 applied at 0.5% significantly enhanced pepper N and K use efficiencies (Figs. 4A and 4C). YD2 application showed no significant effect on P use efficiency (Fig. 4B).

**Figure 4:**
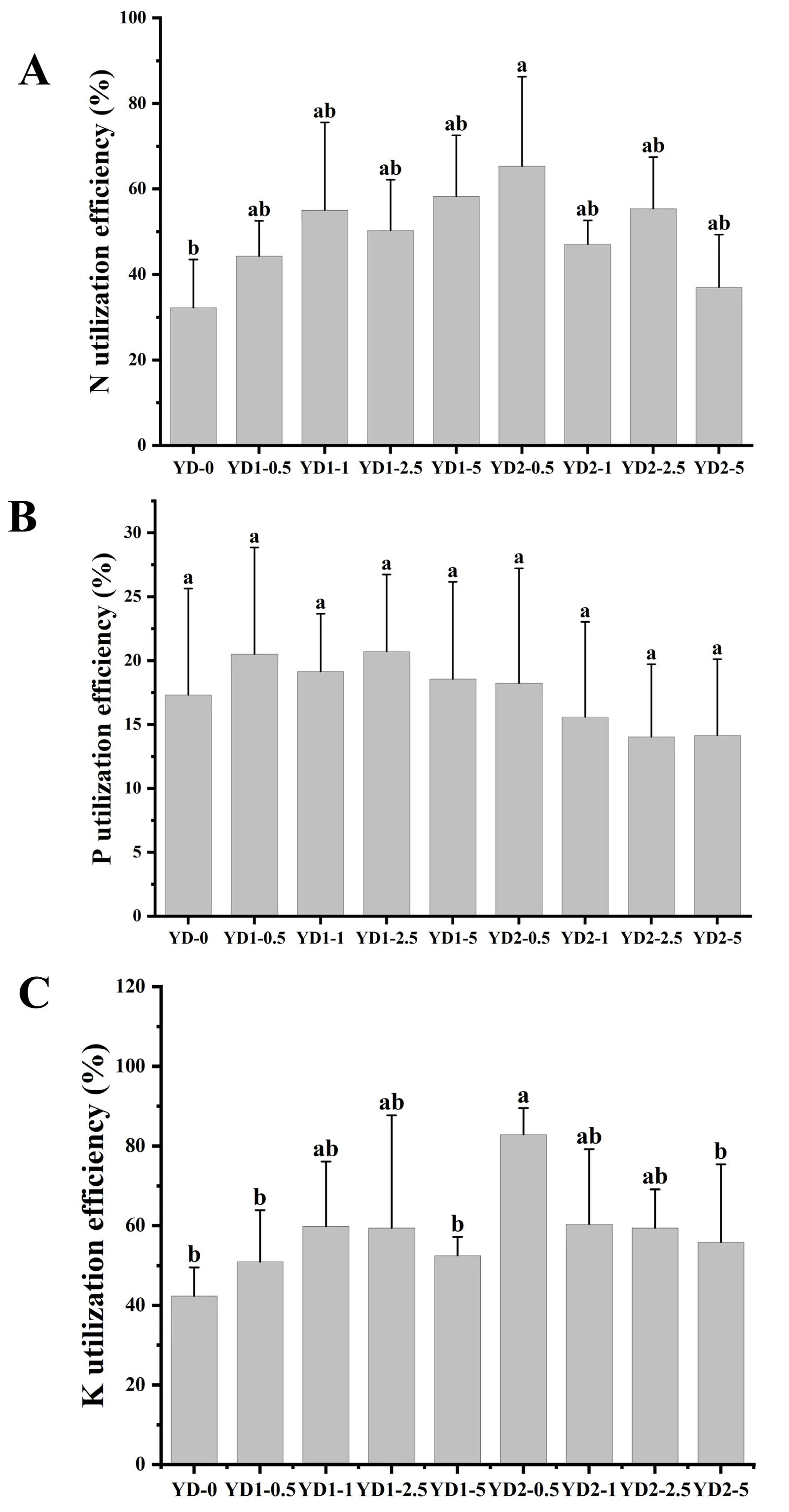
The effect of YDs on fertilizer utilization efficiency in chili pepper. (A) nitrogen N, (B) phosphorus P, (C) potassium K.

### Effects of YD on physiological indicators in pepper under low temperature stress

To investigate the physiological and biochemical changes in chili pepper roots and the structural shifts in the rhizosphere microbial community under low-temperature stress, a pot-culture experiment was conducted under controlled conditions. Four key treatments were established: CK (unfertilized control), 0% YD (fertilizer without additives), 1% YD1, and 1% YD2. The objective of this experimental design was to elucidate the key mechanisms by which yeast metabolites enhance cold stress tolerance in pepper. After 15 d of low-temperature exposure, root activity in all treatments except YD2 was markedly lower than that under normal temperature conditions, indicating substantial suppression of root vitality by cold stress. Under low-temperature conditions, YD1 treatment showed no significant difference in root activity compared to CK and 0% YD, whereas YD2 treatment demonstrated significantly higher root activity than all other treatments, revealing that 1% YD2 effectively maintains root function during low temperature stress (Fig. 5A). Under normal conditions, the application of 1% YD1 and YD2 had no effect on the chlorophyll content. Under low temperature stress, application of 1% YD1 and YD2 exhibited significantly higher chlorophyll content than control, indicating YDs capacity to mitigate cold-induced chlorophyll degradation (Fig. 5B). Under both low-temperature and normal-temperature conditions, fertilizer application significantly reduced soluble sugar content in pepper fruits compared to the control without fertilizer application (CK), Under low-temperature stress, the soluble sugar and proline content in leaves increased compared to the normal-temperature, unfertilized control (NT-CK). However, when compared specifically to the low-temperature, fertilized control (LT-YD-0), these increases were not statistically significant for any of the YD treatments (Figs. 5C and 5D). As expected, low-temperature stress itself significantly increased the activities of SOD and POD across all treatments when compared to their respective normal-temperature counterparts (Figs. 5E, 5F). Within the low-temperature condition, application of 1% YD1 or 1% YD2 further significantly enhanced the activities of both enzymes compared to the low-temperature fertilized control (LT-YD-0).

**Figure 5:**
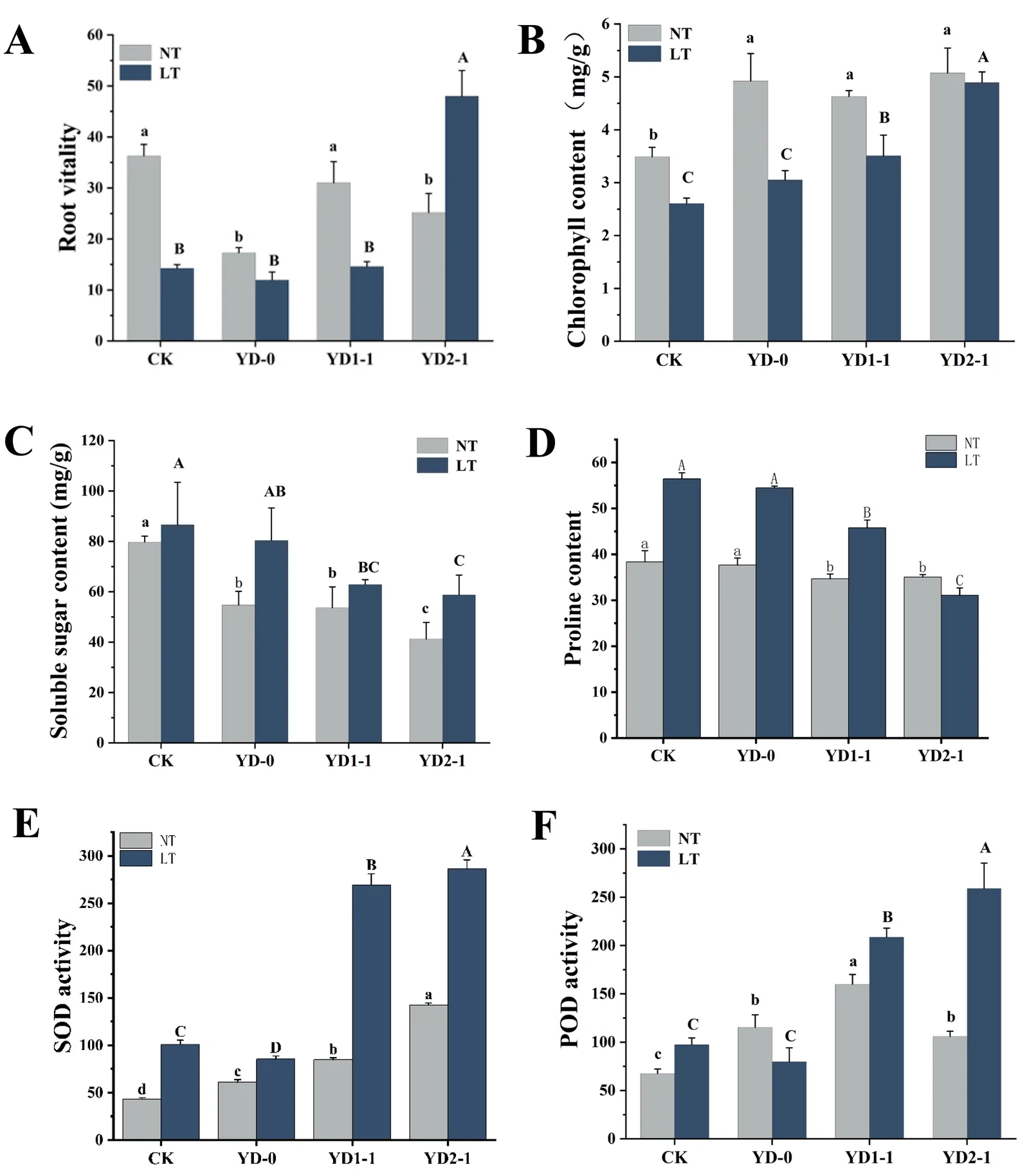
Effect of YDs on physiological indicators under normal and low temperature conditions. (A) root activiey; (B) chlorophyll content; (C) soluble suger content; (D) proline content; (E) SOD activity; (F) POD activity; NT, normal temperature; LT, low temperature.

### Effects of YD on rhizosphere microbial communities

Under normal temperature conditions, principal coordinate analysis (PCoA) revealed that the rhizosphere bacterial community structures of the two YD treatments (YD1-1 and YD2-1) were clearly separated from that of the control group (YD-0) along the first principal coordinate (PC1) (Fig. 6A). PC1 explained 42.09% of the total variation. Permutational multivariate analysis of variance (PERMANOVA) confirmed a statistically significant difference among the treatment groups (*R*^2^ = 0.8930, *p* = 0.007). This indicates that YD application significantly altered the rhizosphere bacterial community composition under optimal growth conditions.

**Figure 6:**
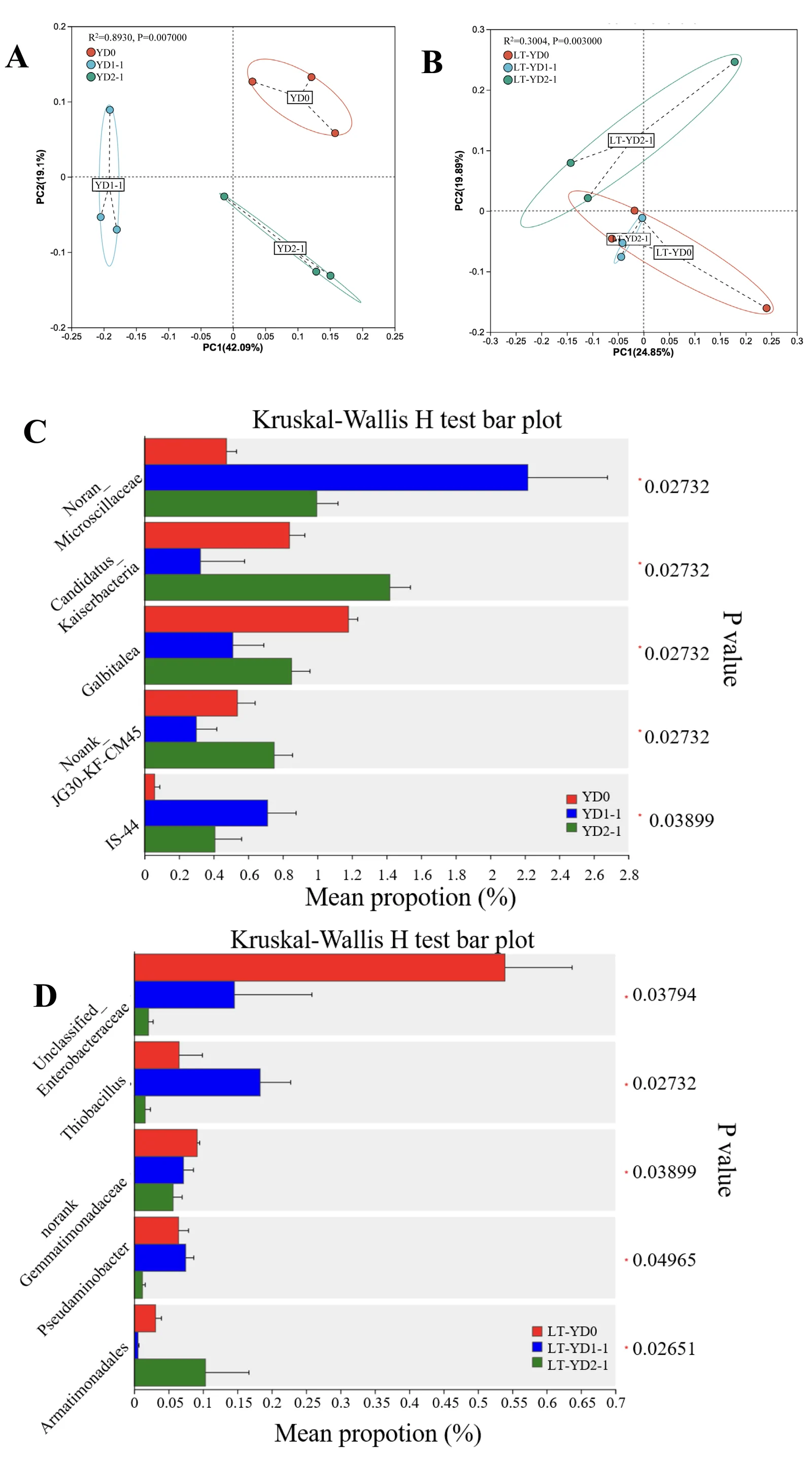
Effects of YDs on rhizosphere bacterial community structure under normal and low-temperature stress conditions. (A, C) normal temperature; (B, D) low temperature.

Under low-temperature stress, the separation between the YD treatments (LT-YD1-1, LT-YD2-1) and the control (LT-YD-0) in the PCoA plot was diminished, with partial overlap observed among samples (Fig. 6B). PC1 accounted for 24.85% of the total variation, a lower proportion than under normal temperature. The PERMANOVA result remained significant but showed a reduced effect size (*R*^2^ = 0.3004, *p* = 0.003). This pattern suggests that the regulatory effect of YDs on bacterial community structure was less distinct under cold stress. We interpret this as an associative observation; a formal statistical test for a temperature-by-treatment interaction on community structure was not performed, so we refrain from concluding a definitive attenuation of YD regulation.

Analysis of differentially abundant taxa showed that under normal temperature, the YD1-1 treatment was associated with a higher relative abundance of unclassified *Microscillaceae* and the IS-44 lineage, while the YD2-1 treatment was associated with a higher relative abundance of *Kaiserbacteria* (Fig. 6C). Under low-temperature stress, the YD1-1 treatment was associated with a higher relative abundance of *Thiobacillus*, while YD2-1 was associated with a higher relative abundance of unclassified *Armatimonadales* (Fig. 6D). Notably, both YD treatments were associated with a lower relative abundance of *Enterobacteriaceae* in the pepper rhizosphere under both temperature conditions.

## Discussion

Our study demonstrates that yeast derivatives (YDs), particularly YD2, can modulate pepper growth, nutrient use efficiency, physiological responses to low-temperature stress, and rhizosphere bacterial community structure. However, the observed effects likely arise from a combination of factors inherent to the YDs, and their interpretation requires careful consideration of correlation *versus* causation.

The superior performance of YD2 under normal temperature conditions—evidenced by enhanced yield, biomass, and N/K use efficiency—cannot be solely attributed to its distinct phytohormone profile (higher IAA, ACC, and SA). It is crucial to acknowledge that YD2 also possessed substantially higher macronutrient (N, P, K) content than YD1 (Table 1). Therefore, the observed benefits likely result from the combined contribution of nutritional supplementation and hormonal stimulation, rather than from hormonal regulation alone. This integrated perspective is essential for accurately understanding the mode of action of complex biostimulants like YDs. The more limited effects of YD1 may reflect its overall lower nutrient content and different hormonal composition.

Under low-temperature stress, YD2 application helped maintain root activity and chlorophyll content, and increased the activities of SOD and POD (Fig. 5). While these physiological adjustments are commonly associated with improved stress tolerance (Alici & Arabaci, 2016; Ignatenko et al., 2021), we interpret these findings with caution. Enhanced antioxidant enzyme activity can indicate a more robust cellular defense mechanism, but it may also simply reflect a heightened level of oxidative stress. In the absence of direct measurements of reactive oxygen species (ROS) or membrane damage, our data support an association between YD2 and activated antioxidant systems, but does not conclusively prove enhanced protection. Similarly, the observed increases in soluble sugar and proline content under low temperature across treatments (Figs. 5C, 5D) suggest a general stress acclimation response (Yang et al., 2020; Yuanyuan et al., 2009). Our results indicate that YD treatments did not uniquely alter these osmolyte levels compared to the stressed control, underscoring the need to interpret these common biomarkers in direct relation to the specific treatment effects observed in our experiment.

A critical and somewhat paradoxical finding is that while YD2 improved several physiological parameters under low-temperature stress, it did not translate into a significant increase in yield at any application rate (Fig. 2). This disconnect suggests that the short-term (15-day) physiological improvements induced by YD2 under our pot-based conditions were insufficient to overcome the severe growth limitation imposed by cold stress, or that yield determinants other than those measured were compromised. This highlights a key distinction between stress tolerance mechanisms at the biochemical level and ultimate agronomic productivity, emphasizing that biostimulant efficacy under stress is context-dependent and may not always confer yield advantages.

The regulatory effects of YDs on rhizosphere bacterial community structure were evident under normal temperature but attenuated under low-temperature stress (Fig. 6). We interpret these shifts as associations rather than established functional outcomes. For instance, the enrichment of taxa such as *Microscillaceae* (YD1) and *Kaiserbacteria* (YD2) under normal temperature, or Thiobacillus (YD1) and *Armatimonadales* (YD2) under stress, suggests that YDs can selectively influence microbial populations. However, given the broad taxonomic categories identified and the lack of functional validation in our study, we refrain from assigning specific ecological roles (*e.g.*, nutrient cycling, pathogen suppression) to these taxa based solely on literature from other systems (Ahmed et al., 2023; Heydari & Pirzad, 2020; Shen & Lin, 2021). Similarly, the decreased relative abundance of Enterobacteriaceae is noted, but we acknowledge that this family encompasses immense functional diversity, including non-pathogenic members. Thus, this change cannot be reliably interpreted as a biocontrol effect without direct evidence. Our microbial conclusions are strictly correlative, and future work involving metagenomics or culturing is needed to establish causative links between YD-induced microbial shifts and plant phenotypic outcomes.

Finally, we recognize several important limitations of our study. The experiments were conducted under controlled pot conditions, which simplify soil-plant interactions and may not fully represent field complexity. The low-temperature stress was applied for a relatively short duration (15 days), and replication was limited for some resource-intensive assays. Most importantly, the findings require validation under field conditions to assess the practical agronomic value and sustainability of YD application. It should be noted that the significant effects of YD on yield were observed at only a subset of application rates (primarily 1% YD2 under NT), and these findings should be confirmed in future replicated studies before being generalized. Despite these limitations, our results provide a foundation for understanding the multifaceted effects of yeast derivatives on pepper and underscore the need for an integrated perspective that considers both nutrient and non-nutrient components when evaluating biostimulant function.

## Conclusions

The study demonstrates that the two YDs (YD1 and YD2) can enhance chili pepper yield under normal temperature conditions, primarily through different mechanisms. YD2 which was characterized by high auxin content exerted a more pronounced promoting effect on yield and nutrient use efficiency. In contrast, YD1, which is enriched in cytokinins, did not significantly improve nutrient use efficiency, though it may exhibit other growth-promoting effects. Under low temperature stress, the beneficial effects of these YDs were less attenuated; however, YD2 still showed potential in maintaining root activity and enhancing the antioxidant defense system of pepper plants. The regulatory effect of YDs on rhizosphere bacterial community structure under normal temperature conditions suggests that these derivatives may promote plant growth through indirect pathways involving rhizosphere microbial interactions.

## Supplemental Information

10.7717/peerj.21604/supp-1 Supplemental Information 1 Fertilizer utilization rate

10.7717/peerj.21604/supp-2 Supplemental Information 2 Physiological indicators of chili pepper under normal and low temperature-raw data

10.7717/peerj.21604/supp-3 Supplemental Information 3 Yield of pepper under normal and low temperature

## Additional Information and Declarations

### Data Availability

The following information was supplied regarding data availability: The raw measurements are available in the Supplemental Files. The raw sequence data are available at NCBI BioProject: PRJNA1395681.
